# Comparative Effectiveness of Local Application of Chlorhexidine Gluconate, Mupirocin Ointment, and Normal Saline for the Prevention of Peritoneal Dialysis-related Infections (COSMO-PD Trial): a multicenter randomized, double-blind, controlled protocol

**DOI:** 10.1186/s13063-019-3953-8

**Published:** 2019-12-19

**Authors:** Surapon Nochaiwong, Chidchanok Ruengorn, Kajohnsak Noppakun, Setthapon Panyathong, Phongsak Dandecha, Manish M. Sood, Chalermpong Saenjum, Ratanaporn Awiphan, Sasithorn Sirilun, Pajaree Mongkhon, Wilaiwan Chongruksut, Kednapa Thavorn, for the Thai Renal Outcomes Research (THOR) Investigators

**Affiliations:** 1 Department of Pharmaceutical Care, Faculty of Pharmacy, Chiang Mai University, Chiang Mai, 50200 Thailand; 2 Pharmacoepidemiology and Statistics Research Center (PESRC), Chiang Mai University, Chiang Mai, 50200 Thailand; 3 Division of Nephrology, Department of Internal Medicine, Faculty of Medicine, Chiang Mai University, Chiang Mai, 50200 Thailand; 4 Kidney Center, Nakornping Hospital, Chiang Mai, 50180 Thailand; 5 Division of Nephrology, Department of Internal Medicine, Prince of Songkla University, Hat Yai, Songkhla, 90110 Thailand; 6 Ottawa Hospital Research Institute, Ottawa Hospital, Ottawa, Ontario K1H 8L6 Canada; 7 Division of Nephrology, Department of Medicine, University of Ottawa, Ottawa, Ontario Canada; 8 Department of Pharmaceutical Sciences, Faculty of Pharmacy, Chiang Mai University, Chiang Mai, 50200 Thailand; 9 School of Pharmaceutical Sciences, University of Phayao, Muang, Phayao, 56000 Thailand; 10 Depertment of Surgery, Faculty of Medicine, Chiang Mai University, Chiang Mai, 50200 Thailand; 11 School of Epidemiology and Public Health, Faculty of Medicine, University of Ottawa, Ottawa, Ontario K1G 5Z3 Canada; 12 Institute of Clinical and Evaluative Sciences, ICES uOttawa, Ottawa, Ontario K1Y 4E9 Canada

**Keywords:** Chlorhexidine, Cost-utility, Infection, Mupirocin, Normal saline, Peritoneal dialysis, Prevention, Randomized controlled trial

## Abstract

**Background:**

Current international guidelines recommend the use of a daily topical exit-site antimicrobial to prevent peritoneal dialysis (PD)-related infections. Although nonantibiotic-based therapies are appealing because they may limit antimicrobial resistance, no controlled trials have been conducted to compare topical antimicrobial agents with usual exit-site care for the prevention of PD-related infections among the Thai PD population. We propose a controlled three-arm trial to examine the efficacy and safety of a daily chlorhexidine gluconate-impregnated patch versus mupirocin ointment versus usual exit-site care with normal saline for the prevention of PD-related infections.

**Methods/Designs:**

This study is a randomized, double-blind, multicenter, active-controlled, clinical trial. Adult patients aged 18 years or older who have end-stage kidney disease and are undergoing PD will be enrolled at three PD Centers in Thailand. A total of 354 PD patients will be randomly assigned to either the 2% chlorhexidine gluconate-impregnated patch, mupirocin ointment, or usual exit-site care with normal saline dressing according to a computer-generated random allocation sequence. Participants will be followed until discontinuation of PD or completion of 24 months. The primary study outcomes are time to first PD-related infection (exit-site/tunnel infection or peritonitis) event and the overall difference in PD-related infection rates between study arms. Secondary study outcomes will include (i) the rate of infection-related catheter removal and PD technique failure, (ii) rate of nasal and exit-site *Staphylococcus aureus* colonization, (iii) healthcare costs, and (iv) skin reactions and adverse events. We plan to conduct a cost-utility analysis alongside the trial from the perspectives of patients and society. A Markov simulation model will be used to estimate the total cost and health outcome in terms of quality-adjusted life years (QALYs) over a 20-year time horizon. An incremental cost-effectiveness ratio in Thai Baht and U.S. dollars per QALYs gained will be illustrated. A series of probabilistic sensitivity analyses will be conducted to assess the robustness of the cost-utility analysis findings.

**Discussion:**

The results from this study will provide new clinical and cost-effectiveness evidence to support the best strategy for the prevention of PD-related infections among the Thai PD population.

**Trial registration:**

ClinicalTrials.gov, NCT02547103. Registered on September 11, 2015.

## Background

Peritoneal dialysis (PD) is a well-established treatment modality of home renal replacement therapy (RRT) for end-stage kidney disease (ESKD) patients and has been available in Thailand for more than three decades. Although technical innovations and improvements in PD practice have significantly decreased PD-associated adverse outcomes, PD-related infections, including PD-related peritonitis and exit-site/tunnel infection, are still the most common complications [1–3]. From the patient/caregiver and healthcare professional’s perspectives, PD-related infection is the highest priority outcome in PD treatment [4, 5]. Moreover, PD-related peritonitis is the leading cause of long-term structural and functional peritoneal membrane malfunction [1, 6]. As a result, substantial PD technique failure, hospitalizations, mortality, and healthcare costs are responsible for the limited modality options as an RRT and limited long-term PD use [1–3, 6].

Several strategies involve topical antimicrobial agents to prevent PD-related infections and are being widely used [7]. Of these, daily exit-site care with mupirocin cream or ointment is the most common approach, targeting the exit-site infections caused by *Staphylococcus aureus*. This strategy has been proven to be effective by several existing observational studies, randomized controlled trials (RCTs), and meta-analyses and is recommended by the International Society for Peritoneal Dialysis (ISPD) guidelines [7–13]. Regarding antimicrobial activity, mupirocin is only effective against gram-positive organisms, primarily *S. aureus*. Nevertheless, the emergence of mupirocin resistance has been reported in the long-term application of exit-site care in recent years [14–16].

Chlorhexidine is a water-soluble cationic biguanide with broad-spectrum properties, including antimicrobial effects against gram-positive and gram-negative bacteria, facultative anaerobes and aerobes, and yeast, and it inactivates some viruses [17]. Recently, several controlled trials have revealed that preoperative skin preparation and skin decolonization with chlorhexidine gluconate decreases the rates of surgical-site infections, healthcare-associated infections and bacterial transmission in noncritically and critically ill patients [18–21]. Among PD patients, topical 0.05% to 4% chlorhexidine gluconate aqueous solution, with or without isopropyl alcohol, also commonly has been used in previous practice [22–24] and is recommended by the ISPD guidelines [7]. Chlorhexidine is safe and poorly absorbed through intact adult skin [17]. Nevertheless, the daily application of chlorhexidine gluconate for exit-site care may induce local skin irritation, which could limit patient acceptability and long-term compliance. Whether local irritability limits the long-term and widespread use of chlorhexidine gluconate remains uncertain.

In addition to the ISPD recommendations, two comparative observational studies [25, 26] and one RCT [22] have suggested that exit-site care with normal saline is well tolerated and may be an alternative strategy for the prevention of PD-related infections. The results of these studies revealed that normal saline was not superior or more beneficial than other agents for preventing exit-site infections. However, evidence of exit-site care with normal saline dressing was insufficient and remains unclear.

To date, few RCTs have identified the optimal antimicrobial agent or alternative strategies for the prevention of PD-related infection. Although antimicrobial resistance with routine use is a concern, no RCTs have compared topical antimicrobial agents with usual exit-site care for the prevention of PD-related infections among the Thai PD population. In the COSMO-PD (Chlorhexidine glucOnate verSus Mupirocin Ointment in the prevention of Peritoneal Dialysis-related infection) trial, we aim to assess the safety, efficacy, and cost-effectiveness of a chlorhexidine gluconate-impregnated patch, mupirocin ointment, and usual exit-site care with normal saline dressing in preventing PD-related infections. We hypothesize that the chlorhexidine gluconate-impregnated patch or mupirocin ointment will be superior to usual exit-site care with normal saline in terms of the efficacy and cost utility of the interventions.

## Methods/Design

### Trial design

**Fig. 1:**
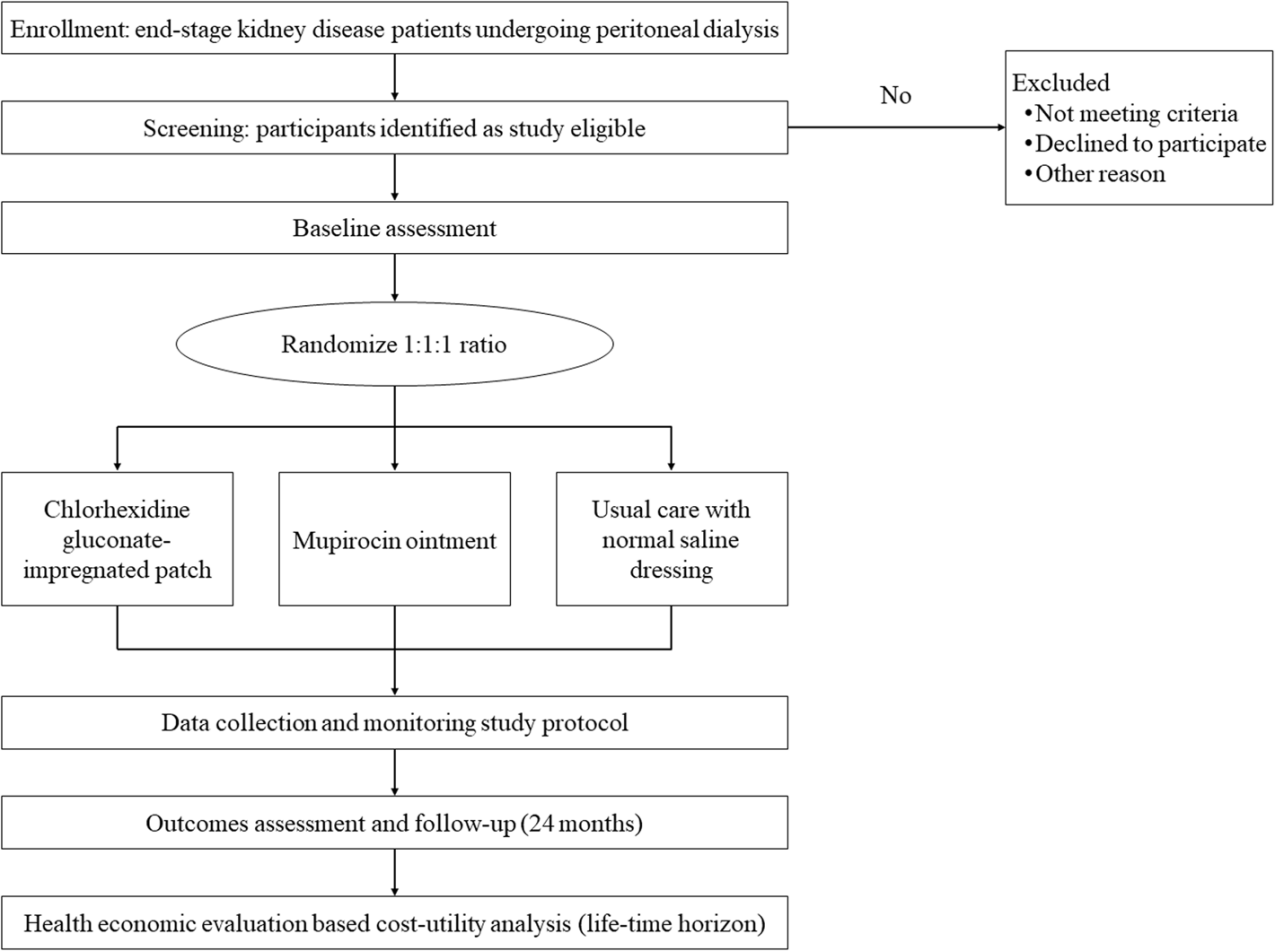
Study flow of the COSMO-PD trial

The COSMO-PD is an active-controlled, double-blinded, multicenter, randomized clinical trial with usual exit-site care. This trial has been registered with ClinicalTrials.gov (NCT02547103). The study protocol was drafted in accordance with the Standard Protocol Items: Recommendations for Intervention Trials (SPIRIT) statement (Additional file 1) [27]. The study flow is illustrated in Fig. 1.

### Trial population and study setting

Adult PD patients, 18 years or older, from three settings in Thailand will be screened for eligibility, including from (i) Nakornping Hospital, Chiang Mai, the largest PD program in Northern Thailand; (ii) Maharaj Nakorn Chiang Mai Hospital, Chiang Mai University, University Hospital of Northern Thailand; and (iii) Songklanagarind Hospital, Prince of Songkla University, University Hospital of Southern Thailand. The inclusion and exclusion criteria are provided in Table 1.

**Table 1:** Eligibility criteria of the COSMO-PD trial

Inclusion criteria	Exclusion criteria
• Participants aged 18 years or over at the date of screening • Participants with ESKD who were undergoing PD by either automated peritoneal dialysis or continuous ambulatory peritoneal dialysis	• History of psychological illness or condition that interferes with the ability to understand or comply with the requirements of the study • Recent (within 1 month) exit-site/tunnel infection or PD-related peritonitis • Known hypersensitivity to or intolerance of chlorhexidine or mupirocin • Current or recent (within 1 month) treatment with antibiotics administered by any route • Nasal or exit-site carriage of mupirocin-resistant *S. aureus* or chlorhexidine-resistant *s. aureus*

Abbreviations: *ESKD* end-stage kidney disease, *PD* peritoneal dialysis, *S. aureus Staphylococcus aureus*

### Patient recruitment

We will pre-screen all PD patients for eligibility. The research team will approach all eligible patients at each PD center and identify patients who are interested in participating in the study. After recruitment and informed consent, participants and their caregivers (if available) will undergo screening to determine *S. aureus* colonization. Screening cultures will be performed by trained study team members using standardized methodology by obtaining a nasal swab from participants and their main caregivers. Participants will also be screened for *S. aureus* colonization at the exit site. All sample screening cultures will be analyzed at the Microbiology Laboratory, Faculty of Pharmacy, Chiang Mai University, according to the Clinical and Laboratory Standards Institute guidelines. Recruitment will occur continuously over a 3-year period to meet the target participant population.

### Interventions

All participants will be required to perform daily washings of the exit site with antibacterial soap while showering, followed by drying of the exit site using a sterile gauze pad. Once-daily usual exit-site care dressing with normal saline will be performed for all participants before application of the randomly assigned fixed-order interventions, as follows: (i) chlorhexidine gluconate-impregnated patch plus placebo ointment base or (ii) placebo sterile patch plus mupirocin ointment or (iii) both placebo sterile patch and placebo ointment base. Participants who were *S. aureus* nasal carriers (within 4 weeks in the screening period) will be decolonized with 2% mupirocin ointment applied to both anterior nares twice daily for 5 consecutive days before the trial starts. All medications administered as a part of standard PD care will be allowed. Details of the investigational medicinal products (IMPs) and their administration are provided in Table 2 and Additional file 2: Appendix 1. Participants will record the use of IMPs with adherence, which will be monitored by their return of the relevant packaging (plastic sachets and ointment tubes).

**Table 2:** IMPs in the COSMO-PD trial

Intervention	Chlorhexidine gluconate	Mupirocin ointment	Usual care with normal saline dressing
Description of IMPs	Non-ionized cloth impregnated with 2% chlorhexidine gluconate (Ion PAD PLUS CHG2®, Pose Health Care., Ltd.)	2% mupirocin calcium ointment (Charoon Bhesaj Co., Ltd.)	Isotonic solution, 0.9% sodium chloride irrigation USP (A.N.B. Laboratories Co., Ltd.)
Packaging	Non-rinse, disposable, single use in plastic sachet	5-g ointment tube	Plastic bottle container 500 mL, sterile and non-pyrogenic
Dummy placebo	Identical placebo ointment tube	Identical plastic sterile non-ionized patch sachet	Identical both placebo ointment tube and placebo plastic sterile non-ionized patch sachet
Administration	Wiping over PD catheter exit site after dressing with normal saline solution, then applying with placebo ointment base by participants or their caregivers, once daily.	Daily applying to PD catheter exit site by participants or their caregivers after dressing with normal saline and wiping with placebo sterile non-ionized patch, respectively	Daily dressing exit site with normal saline solution by participants or their caregivers, then wiping with placebo sterile non-ionized patch and applying with placebo ointment base, respectively
Duration	Over a 24-month period	Over a 24-month period	Over a 24-month period

Abbreviations: *IMPs* investigational medicinal products, *PD* peritoneal dialysis, *USP* the United States Pharmacopeia

### Randomization and allocation concealment

Eligible participants will be randomized with a 1:1:1 allocation ratio to receive the interventions. Randomization will be conducted by a blinded member of the research team using random permuted blocks stratified according to the history of PD-related infections (exit-site/tunnel infection or peritonitis) and the study setting. A list of numbers will be generated by copyrighted software (available at http://www.randomizer.org). The randomization blocks will be blinded to the investigators. Each study setting will be concealed using opaque envelopes opened only upon recruitment of an individual consented participant.

### Blinding

In the COSMO-PD trial, participants and their caregivers, physician, PD nurse, and all staff members at the study site will be blinded. Based on the blinding method, the outcomes of interest and statistical analysis will be assessed by a third-party not participating in recruitment or treatment follow-up. Unblinding will be permitted in cases of serious adverse event or in emergencies that affect optimal treatment care. The investigators will be unblinded only at the end of the study.

Regardless of their study arm allocation, participants will receive a set of two identical ointment tube (either IMPs or placebo) and a plastic nonionized patch sachet (either IMPs or placebo) with directions for use. The study intervention drugs will be administered by nursing staff at each study site. Physicians will access participant eligibility, obtain consent, recruit participants, care for participants during the study, collect data, and assess outcomes.

To examine our blinding procedures, we conducted a pilot study between June 2016 and August 2016 and enrolled 30 pilot participants at two study settings (Nakornping Hospital and Maharaj Nakorn Chiang Mai Hospital). No change was made to the study protocol interventions or administration based on this pilot study. After completion of the pilot study ensuring feasibility of the IMPs process and study flow in August 2016, the actual COSMO-PD trial enrollment was initiated in September 2016.

### Participants’ timeline and assessment

Participants will be assessed every 3 months from baseline to 24 months alongside routine outpatient PD clinic visits as per the trial schedule for assessment (Table 3). During the assessment period, participants and their assisted caregivers (if available) will be screened for nasal and PD catheter exit-site *S. aureus* colonization. Sociodemographic data (e.g., age gender, smoking and alcohol drinking status, weight, height, PD vintage, cause of ESKD, residual urine volume, dialysate adequacy, and routine laboratory tests) and details of medical history and medications will be gathered at baseline. Dialysate characteristics and exit-site assessment, using Twardowski and Prowants’ classification system [28], will be assessed at each visit as well as adverse events and skin reactions.

**Table 3:** Schedule of observation and procedures

Parameter	Study period: time (month)											
	Screening	Assessment and follow-up									Close-out	
	-1	0	3	6	9	12	15	18	21	24	25	26
Check eligibility against inclusion/exclusion criteria and medication review	X											
Screening for *S. aureus* colonization	X			X		X		X		X		
Gartering informed consent and randomization		X										
Sociodemographic and lifestyle data^a^		X										
Medical history by Charlson comorbidity index and ESKD etiology		X										
Physical examination	X	X	X	X	X	X	X	X	X	X		
PD-related infection: dialysate characteristics and exit-site assessment		X	X	X	X	X	X	X	X	X		
Routine laboratory tests (performed locally)^b^		X	X	X	X	X	X	X	X	X		
Dialysate adequacy and peritoneal equilibration test		X				X				X		
HRQOL and mental health^c^		X		X		X		X		X		
Record medication changes		X	X	X	X	X	X	X	X	X		
Safety profiles: skin reactions and adverse events documentation		X	X	X	X	X	X	X	X	X	X	
Hospitalization and emergency visit		X	X	X	X	X	X	X	X	X		
Assess adherence with trial treatment allocation and other medication^d^			X	X	X	X	X	X	X	X		
Healthcare costs: direct medical cost, direct non-medical cost, and indirect cost		X				X				X		
Data monitoring			X	X	X	X	X	X	X	X		
Statistical analysis and reporting											X	X

^a^To include date of birth, date of PD initiation, gender, weight, body mass index, marital status, education, income, insurance, smoking and alcohol status

^b^To include complete blood count, biochemistry, liver function test, and dialysate profile parameter

^c^To include the Kidney Disease Quality of Life-36, EuroQol-5 dimension-5 level, and BDI-II, Beck Depression Inventory-II questionnaires

^d^To include the visual analog scale-medication adherence and the medication-taking behavior measure for Thai patients questionnaires

Abbreviations: *ESKD* end-stage kidney disease, *HRQOL* health-related quality of life, *PD* peritoneal dialysis, *S. aureus Staphylococcus aureus*

### Outcomes

#### Primary outcome

The primary outcomes of the COSMO-PD trial are time-to-first PD-related infection (exit-site/tunnel infection or peritonitis) event and overall difference in PD-related infection rates between study arms. PD-related peritonitis and exit-site/tunnel infection will be defined according to the ISPD guidelines [2, 7]. Participants will be diagnosed as having peritonitis if they meet at least two of the following criteria: (i) clinical features (e.g., abdominal pain and/or cloudy dialysis effluent); (ii) dialysate effluent white cell count more than 100 cell/μL (after a dwell time of at least 2 h), with polymorphonuclears making up more than 50%; and (iii) positive dialysis effluent culture. Exit-site infection and tunnel infection will be indicated as the presence of purulent discharge (with or without erythema of the skin) and evidence of collection along the catheter tunnel (clinical inflammation or ultrasonographic), respectively.

#### Secondary outcomes

Secondary outcomes include the following:

*Clinical events*

- Infection-related catheter removal
- PD technique failure

*S. aureus colonization*

- Incidence of nasal *S. aureus* colonization
- Incidence of exit-site *S. aureus* colonization

*Healthcare costs*

- Direct medical costs including for the IMPs, non-IMPs and equipment, outpatient and emergency visits, hospitalization, antimicrobial treatment for PD-related infection, laboratory tests and procedures, and costs related to adverse events
- Direct non-medical costs including for transportation and the monetary value of informal care
- Indirect costs including daily wages of participant and family caregiver for treatment follow-ups and extra visits

*Safety*

- Skin reactions
- Safety of IMPs related to potential harm (e.g., adverse events, serious adverse events, participant survival in each group, hospitalization, and emergency visits)

#### Additional outcomes

Additional outcomes include the following:

*Health-related quality of life*

- Kidney Disease Quality of Life-36, which is a kidney-specific quality-of-life instrument that explores generic core plus burden of kidney disease, symptoms/problems of kidney disease, and effects of kidney disease scales [29]
- EuroQol-5 dimension-5 level (EQ-5D-5 L), which is a 5-level assessment of mobility, self-care, usual activities, pain/discomfort, anxiety/depression, and the visual analog scale [30]

*Depressive symptoms*

- Beck Depression Inventory-II, which is a 21-item self-reporting questionnaire for evaluating the severity of depression in normal and psychiatric populations [31]

*Medication adherence*

- Direct observation and recording of the use of IMPs via return plastic sachets and ointment tubes
- Global rating of medication adherence by the visual analog scale, where the self-reported adherence level is illustrated with a 10-cm line, where 0 represents “non-adherence—none of the medications taken” and 10 represents “good adherence—every single medication consistently taken”
- Medication-taking behavior measure for Thai patients, which includes a 6-item assessment of mediation-taking behaviors among Thai patients with chronic diseases [32]

### Safety monitoring and trial-related injury

The independent multidisciplinary Data Safety Monitoring Board (DSMB) will be assembled to oversee the study in terms of monitoring and evaluating safety and trial-related injury. Any adverse event needs to be documented in detail, including information on the starting point of symptoms, participant symptoms, severity, duration of the condition, any management administered, final outcome, and relationship with the IMPs among others. In the case of a serious adverse event, the investigator is responsible for informing the DSMB and contacting the statistician to obtain the participant’s allocation information immediately after he/she has established the event.

Additionally, skin reactions will be closely monitored, and participants will be asked to shade the parts of the body to scale if involved. The skin-related event will be classified into Grade I (faint macular erythema [redness] only), Grade II (erythema, edema, and possibly papules), Grade III (erythema, edema, papules, and blisters), and Grade IV (blisters and ulceration [skin breakdown]).

No clinical trial insurance will be provided, and participants will not receive financial compensation for any trial-related injury. However, participants will receive full access to outpatient and in-hospital standard of care.

### Study auditing

A clinical monitor will visit the study sites every 2 weeks to check the progress of the study. Important points to be checked include whether the investigator has conducted the study as per protocol, how many participants were screened and enrolled, and if all eligible participants signed the informed consent form. Completeness of case report form and other essential documents, as well as records of any drop-outs or adverse events, will be checked for correctness and consistency with the source documents in a timely manner.

### Statistical analysis plan

#### Sample size and power calculations

Sample size calculation was based on information from the Thai Renal Outcomes Research-Peritoneal Dialysis database collected between 2006 and 2016, with an estimated rate of 0.31 episodes per patient-year and 0.39 episodes per patient-year for exit-site infection and peritonitis, respectively (composite rate of PD-related infections of 0.70 episodes per patient-year) [3]. To detect an expected clinically relevant difference in both exit-site infection and peritonitis rate of 30% (0.70 episodes per patient-year vs. 0.49 episodes per patient-year) between our usual exit-site care with normal saline and interventions (either chlorhexidine gluconate-impregnated patch or mupirocin ointment), approximately 118 participants per group will be enrolled to obtain 80% power with a two-sided significance level of 0.05, while allowing for 25% all-cause dropout during the study period. Thus, the overall targeted minimum sample size will be 354 participants.

#### Data management and monitoring

A paper-based case report form will be composed before the study commences. Each variable is carefully coded for auditing and statistical analysis. The participants’ general information will be recorded in the case report form by the responsible investigator, whereas participant-reported information will be documented in the case report form by participants.

We will adopt a double data entry and double check approach to data management. All steps involved in the approach to data management will be independently conducted by two data administrators from the Pharmacoepidemiology and Statistics Research Center. If any inconsistency is identified in the data entry or logic consistence check, the investigators will be contacted for further information and clarification. To protect privacy, participants’ identification information (name, telephone, home address, and participants’ family member information) will not be used with the data management software.

The DSMB will review the analyses in terms of efficacy and safety. While the trial is ongoing, DSMB members have access to original data but are blinded to participant allocation. The principle role of the DSMB is to provide a written recommendation in a timely manner to the investigator to discontinue a trial following discussion and assessment of efficacy and safety data.

#### Statistical analysis

Baseline characteristics will be summarized as number (percentage) or mean ± standard deviation, or medians with interquartile range as suitable. Differences between treatment groups will be compared using Fisher’s exact test and analysis of covariance (ANCOVA) or Kruskal-Wallis test for categorical and continuous variables, respectively. All analyses will be conducted on both a per-protocol and intention-to-treat basis. All analyses will be performed using Stata software version 14.0 (StataCorp, LP) and Microsoft Excel version 2016. They will be two-tailed, and a *P*-value less than 0.05 will be considered statistically significant.

For a primary analysis, Cox proportional hazards regression and Poisson regression analyses will be performed to assess the effectiveness of the chlorhexidine gluconate-impregnated patch and mupirocin ointment compared to usual exit-site care with normal saline for the first episode of PD-related infection and longitudinal rates, respectively. Secondary analysis using statistical methods as described above will then be analyzed to evaluate the effect on the prevention of PD-related infections between chlorhexidine gluconate-impregnated patch and mupirocin ointment. The treatment effects will be estimated as hazard ratios or incidence rate ratios with corresponding 95% confidence interval. Kaplan-Meier survival curves will be constructed for visual presentation of time-to-event comparisons.

Several a priori subgroup analyses with respect to the minimization variables for both primary and secondary outcomes will be investigated. For instance, the lists of variables include age (<65 vs. ≥65 years), gender, history of diabetes, history of PD-related infections (peritonitis or exit-site/tunnel infection), *S. aureus* colonization status (nasal or exit-site PD catheter), PD modality (continuous ambulatory peritoneal dialysis vs. automated peritoneal dialysis), PD vintage, and serum albumin (< 3.5 vs ≥3.5 mg/dL).

To address robustness of the treatment effects, further sensitivity analyses of the primary analysis will be performed by (i) adjusting for the several baseline covariates that seem to affect the outcomes (age, history of diabetes, history of PD-related infections, *S. aureus* colonization status, PD modality, PD vintage, and serum albumin); (ii) restricting the analysis to only PD-related infection episodes with positive cultures to account for misclassification bias; (iii) using the proportional sub-distribution hazards analyses by Fine and Gray method to address the completing risk outcomes, including mode switching to long-term hemodialysis, kidney transplantation, conservative treatment, or death; and (iv) using the multiple imputation method to evaluate the effect estimates when missing data are indicated.

**Fig. 2:**
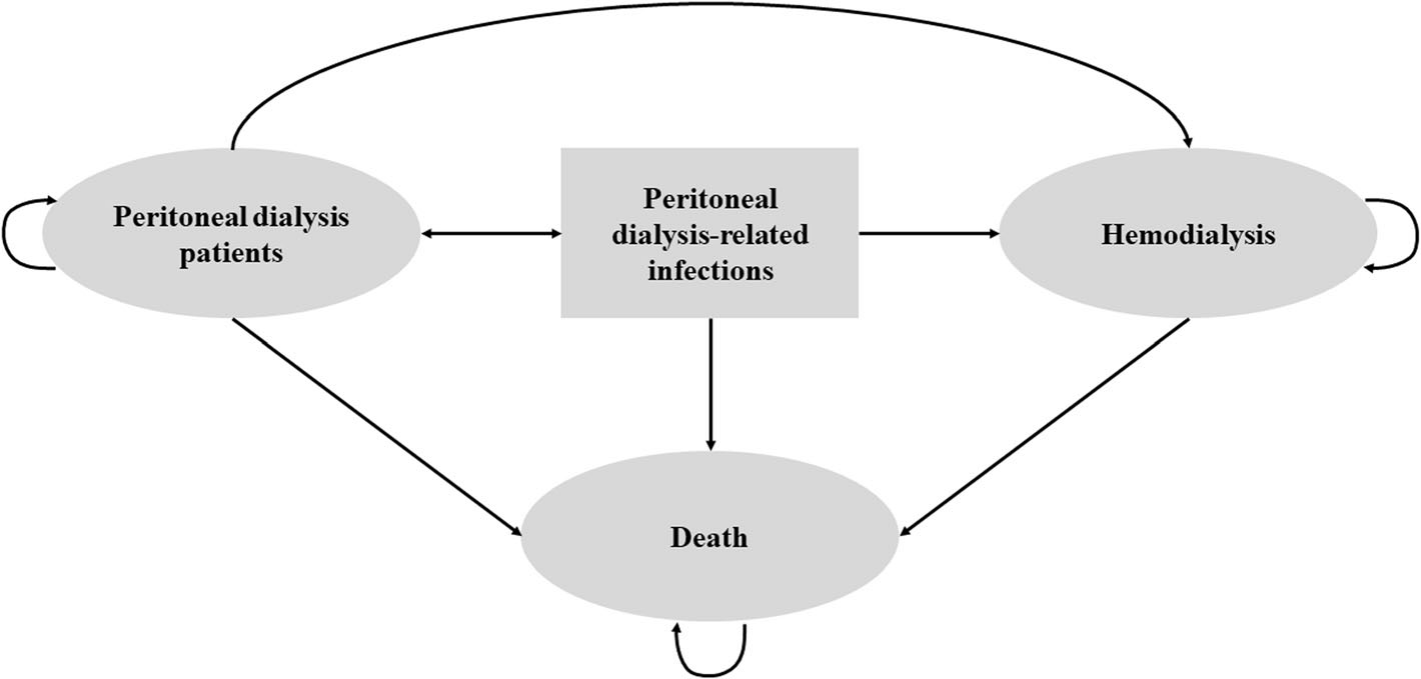
A simplified Markov model structure of the COSMO-PD trial

Moreover, we will conduct a cost-utility analysis alongside the trial. Resource use will be collected using a standard Excel-based costing tool. Healthcare costs will be estimated from patient and societal perspectives and will be expressed in Thai Baht and USD. We will consider direct medical costs, direct nonmedical costs, and indirect costs. Utility values will be estimated for each patient using the EQ-5D-5 L instrument [30]. The EQ-5D-5 L is a five-dimension quality of life instrument designed to elicit utility values for a patients’ current health status. It will be completed at five time points. Responses to the EQ-5D-5 L will be scored using preference weight estimates from Thai populations, [33] which convert the five responses into a single summary index, where a score of one reflects perfect health, and zero is equivalent to dead. We will use a Markov simulation model to estimate the total costs and health outcomes in terms of clinical events and quality-adjusted life years (QALYs) over the patient lifetime (i.e., 20 years). Cost and health outcomes beyond the trial will be derived from a systematic review and from health administrative reports containing information about progression of the disease in Thailand (Fig. 2). As per Thai Health Technology Assessment guidelines, costs and health outcomes will be discounted at an annual rate of 3% [34]. The results of the economic evaluation will be presented as incremental cost-effectiveness ratios comparing the total costs and QALYs among treatment groups. Thai Baht and UDS per QALYs gained will be illustrated. A series of deterministic and probabilistic sensitivity analyses will be performed to evaluate the robustness of the cost-effectiveness estimates and study assumptions. Probabilistic sensitivity analyses results will also be used to create cost-effectiveness acceptability curves, which represent the probability of a treatment being cost-effective to a range of potential threshold values that the decision makers may be willing to pay for an additional unit of effect.

### Ethics and regulatory aspects

The COSMO-PD trial will be conducted according to the standards of the International Conference on Harmonization-Good Clinical Practice. Pharmacovigilance reporting will comply with the 2004 Medicines for Human Use (clinical trials) Regulations and 2006 Amended Regulations. We will conduct the investigation in accordance with the key principles of ethical conduct in research and the Declaration of Helsinki. The sponsors will not be involved with study design, or data assembly and analysis.

#### Informed consent

Participants will be included in the study only if they provide written informed consent. Participant consent will be obtained by trained research assistants (Additional file 2: Appendix 2). Written informed consent will be provided by all participants prior to randomization. If a participant is illiterate, a thumbprint will be required on the consent form in line with the International Conference on Harmonization: Thai Guideline for Good Clinical Practice. Informed consent may be withdrawn at any time during the study period and will have no effect on a participants’ clinical management at the study site.

#### Confidentiality

Consented participants will be assigned a screening number and a study identification code number that will be the primary mode of identification throughout the study. Subsequently, unique identifiers will be generated for computer-based data entry and all specimens. All information collected will remain confidential and shared only within the research study team. Initial screening forms, case report forms, and completed identification code number list will be kept in locked files.

#### Healthcare services and reimbursement

Participants unwilling to participate or who cannot be enrolled into the study due to unmet criteria will be referred for standard protocol for exit-site care at each setting. A clear statement will be provided to indicate that the decision not to participate in the trial will not affect subsequent care. Participants in the trial will be reimbursed for their transport to attend all follow-up visits at the study site (500 Thai baht/visit). No other gifts or payments will be offered.

### Study withdrawal

Participants may withdraw from the trial at any time for any reason. Furthermore, participants who meet any of the following conditions will be allowed to withdraw from the trial: (i) refusing to follow the study protocol; (ii) using other IMPs; (iii) pregnancy during the study period; (iv) switching mode to long-term hemodialysis, kidney transplantation, or conservative treatment; and (v) developing serious adverse drug reactions or suspected unexpected serious adverse reactions. According to the intention-to-treat approach, data collected prior to study withdrawal will be considered for participants who discontinue or deviate from the assigned intervention protocols.

### Patient/public involvement and dissemination of results

Participants and the public had no role in the trial design, recruitment, conduct, or monitoring. Our findings will be published in peer-reviewed journals and disseminated through scientific and professional meetings. Written lay summary results will be available to the public. At the end of the trial, the principal investigators will review and finalize the study report and data set. This report will be shared with the Health Systems Research Institute of Thailand. The investigators commit to reporting data as endorsed by the Consolidated Standards or Reporting Trials guidelines: Extension for Reporting of Multi-Arm Parallel-Group Randomized Trials [35, 36] and the Consolidated Health Economic Evaluation Reporting Standards Statement [37] for reporting parallel group randomized trials and health economic evaluation, respectively.

## Discussion

PD utilization and the number of PD centers are rising dramatically in Thailand owing to the “PD First” policy—the national health policy under universal coverage scheme [38, 39]. Globally, current estimates reveal that PD utilization involves more than 272,000 individuals with ESKD, representing nearly 11% of the dialysis population [40]. As the global burden of chronic kidney disease continues to rise, the annual growth rate of PD is estimated to rise in parallel. This growth in PD use is anticipated to be higher than hemodialysis, particularly in low- and middle-income countries with limited access to center-based hemodialysis and/or kidney transplantation [40]. However, considerable variation remains in the use of PD worldwide, which is attributed to factors implicit in the patients, healthcare processes, practitioners, and healthcare policy [41]. For example, PD utilization in countries with non-financial PD-First policies had higher rates of PD utilization than countries that do not promote PD as the first modality [42].

Generally, the Centers for Disease Control and Prevention recommends short-term use of topical mupirocin alone in patients with *S. aureus* [43]. Furthermore, the World Health Organization recommends the use of mupirocin and/or chlorhexidine in patients with methicillin-resistant *S. aureus* with no specified indications or time of use [44]. The ISPD provides consensus guidelines on the prevention of PD-related infections; however, recommendations are based on variable evidence and concerns exist regarding the emergence of resistance to antimicrobial agents used for this purpose [2, 7]. According to the Peritoneal Dialysis Outcomes and Practice Patterns Study, a degree of country- and facility-based variation exists in the use of prophylactic antimicrobial agents, and this variation likely contributes to differing rates of PD-related infections across countries [45]. For instance, data from 170 PD facilities (encompassing over 11,000 patients) from Australia/New Zealand (ANZ), Canada, Thailand, Japan, the United Kingdom (UK), and the United States of America (USA) illustrated that the use of topical exit-site antimicrobial prophylaxis varies across countries, with Japan and Thailand having the lowest proportions at 4% and 28%, respectively. With regard to topical antimicrobial agents, daily exit-site care mupirocin was observed to be the predominant prophylactic strategy in ANZ (56%), Canada (50%), and the UK (47%); meanwhile, exit-site care with aminoglycosides were more common in the USA (72%) [45]. Likewise, wide variations in exit-care were also observed in a French nationwide cohort from 64 PD centers (2540 incidents of PD patients), and topical mupirocin on the exit site was common (range of 1% to 27%) across center-level characteristics [46].

Although the ISPD guidelines recommend the use of daily topical exit-site antimicrobial to prevent PD-related infections (Level 1B), prophylactic antimicrobial agents for routine exit-site care were not always administered, with use varying worldwide from 6% to 96%—including in Thailand (73%) [45, 46]. Because of the emergence of resistance to antimicrobial agents, particularly mupirocin, treatment failure with mupirocin has been reported and has caused significant concern [14, 15]. Moreover, the practitioners and healthcare team perceived a lack of evidence of treatment effectiveness in the Thai PD population, and economic burden may limit medication availability and locally endorsed prophylactic antimicrobial agents for routine exit-site care in PD patients [45]. However, as infection control practices during PD catheter insertion are important for the prevention of PD-related infection [2, 47], all PD settings in this study had a standard protocol in place for the administration of intravenous antibiotics at the time of PD catheter insertion which might contribute to the rate of post-surgery PD-related infections. Given the concern of the emergence of microbiological resistance, the use of routine exit-site antimicrobial prophylaxis should depend on the circumstantial evidence rate of microbiological resistance and rate of PD-related infections, as well as local geographic and patient demographic factors.

At present, no direct head-to head comparison studies of prophylactic strategies for the prevention of PD-related infections have been conducted in Thailand. Taken together, stronger evidence in terms of clinical and cost-effectiveness outcomes is needed to support the best strategy for prevention of PD-related infections among the Thai PD population. As such, this is the first well-controlled trial to compare the safety, effectiveness, and cost-effectiveness of chlorhexidine gluconate-impregnated patch, mupirocin ointment, and usual exit-site care with normal saline dressing, for the prevention of PD-related infection. Given the “PD First” policy in Thailand, most patients are treated with continuous ambulatory peritoneal dialysis (CAPD), which may limit the generalizability of study findings to other PD modalities.

In summary, the COSMO-PD trial will determine whether exit-site care with chlorhexidine gluconate-impregnated patch or mupirocin ointment decreases the risk of PD-related infections compared to standard exit-site care with normal saline dressing. Novel strategies are needed to prevent PD-related infection and alleviate the healthcare costs, while improving long-term survival in PD patients. The findings of this trial could provide the new clinical and cost-effectiveness evidence of the strategy for the prevention of PD-related infections.

### Trial status

Trial protocol version 2–2016, dated May 9, 2016, was used to prepare this manuscript. The COSMO-PD trial is currently in the participant enrollment phase. A total of 288 eligible participants have been randomized as of July 2019. We anticipate that enrollment will be completed in October 2019.

## Supplementary information

**Additional file 1.** SPIRIT 2013 Checklist: Recommended items to address in a trial protocol and related documents.

**Additional file 2: Appendix 1.** Investigational medicinal products. **Appendix 2.** Participants’ information sheet and informed consent form (in Thai).

## Data Availability

Data sharing is not applicable to this article because no datasets were generated or analyzed during the current study. However, data from the study will be made available at the end of the trial, on request. Requests will be subject to approval by the COSMO-PD chief investigator, the advisory Committee, and the relevant ethical bodies.

## Abbreviations

EQ-5D-5 L: EuroQol-5 dimension-5 level
ESKD: end-stage kidney disease
IMPs: investigational medicinal products
ISPD: International Society for Peritoneal Dialysis
PD: peritoneal dialysis
QALYs: quality-adjusted life years
RCTs: randomized controlled trials
RRT: renal replacement therapy
