# Supplementary material for: Comparative Effectiveness of Local Application of Chlorhexidine Gluconate, Mupirocin Ointment, and Normal Saline for the Prevention of Peritoneal Dialysis-related Infections (COSMO-PD Trial): a multicenter randomized, double-blind, controlled protocol
Source: Trials. 2019 Dec 19;20:754. doi: 10.1186/s13063-019-3953-8 (PMC6924023; doi:10.1186/s13063-019-3953-8)
Supplement: Supplementary file 2 — Additional file 2: Appendix 1. Investigational medicinal products. Appendix 2. Participants’ information sheet and informed consent form (in Thai). [file 13063_2019_3953_MOESM2_ESM.pdf]

Supplementary Material

Comparative Effectiveness of Local Application of Chlorhexidine Gluconate, Mupirocin Ointment, and Normal Saline for the Prevention of Peritoneal Dialysis-Related Infections (COSMO-PD): Protocol for a Multicenter Randomized Controlled Trial

Authors: Surapon Nochaiwong (ORCID iD: orcid.org/0000-0003-1100-7171), Chidchanok Ruengorn (ORCID iD: orcid.org/0000-0001-7927-1425), Kajohnsak Noppakun, Setthapon Panyathong, Phongsak Dandecha, Manish M. Sood, Chalermpong Saenjum[8], Ratanaporn Awiphan, Sasithorn Sirilun, Pajaree Mongkhon, Wilaiwan Chongruksut, Kednapa Thavorn; for the Thai Renal Outcomes Research (THOR) Investigators

Correspondence:

1) Surapon Nochaiwong, PharmD, Department of Pharmaceutical Care, Faculty of Pharmacy, Chiang Mai University, Chiang Mai 50200, Thailand, Phone: 66899973365, Fax: 6653222741, Email: surapon.nochaiwong@gmail.com

or

2) Chidchanok Ruengorn, PhD, Department of Pharmaceutical Care, Faculty of Pharmacy, Chiang Mai University, Chiang Mai 50200, Thailand, Phone: 66810222932, Fax: 6653222741, Email: chidchanok.r@elearning.cmu.ac.th

Supplementary Online Content

**Appendix-I**	Investigational medicinal products	S3
**Appendix-II**	Participants' information sheet and informed consent form (in Thai)	S8

Appendix-I Investigational medicinal products.

Food and Drug Administration

Ministry of Public Health, Thailand

CERTIFICATE OF FREE SALE

Ref.No. 1-1-03-02-15-00114 April 30, 2015

It is hereby certified that **Pose Health Care Limited**, **1 Soi Ramintra 107**, **Ramintra Rd.**, **Kannayao**, **Bangkok 10230**, **Thailand** does manufacture medical device in Thailand pursuant to the Medical Device Act 2008.

The following product may be sold in Thailand and exported without restriction.

Name of Product : **Ion PAD PLUS CHG2**

This certificate is issued upon the request of Pose Health Care Limited, Thailand.

Valid until 29 April 2017

(Mrs. Korrapat Trisarnsri)
Senior Pharmacist
Acting Director of Medical Device Control Division
For Secretary-General Food and Drug Administration

Division of Medical Device Control, Food and Drug Administration
88/24 Tivanon Road, Nonthaburi 11000, Thailand
Tel. (662) 590-7148, Fax. (662) 591-8445

MATERIAL SAFETY DATA SHEET

Ion PAD PLUS CHG 2

1- IDENTIFICATION

POSE HEALTH CARE LIMITED
1 SOI RAMINTRA 107 RAMINTRA RD.,
KANNAYAO, BANGKOK 10230 THAILAND
Tel. 0-2917-8681-4 FAX.0-2917-8685
E-mail : posehealthcare@hotmail.com
www.posehealthcare.com

2- COMPOSITION INFORMATION

	%	CAS#
**Chlorhexidine Gluconate**	**2.0**	**18472-51-0**
**Other innert ingredients and DI. water qs. to**	**100.0**	

3- PHYSICAL AND CHEMICAL PROPERTIES

Boiling Point: N/A
Specific Gravity (H_2O = 1): N/A
Vapor Pressure (mm Hg): N/A
Vapor Density (AIR = 1): Heavier than water
Evaporation Rate (Butyl Acetate = 1) : Slower than ether
Solubility in Water: 100%
Appearance and Odor : Impregnated - Medical absorbent fabric
pH : 5.5 – 7.0
Acute oral toxicity (LD50) of : 13,929.97 mg/kg [Rat.].

4- FIRE AND EXPLOSION HAZARD DATA

Flash Point (Method Used): N/A
Flammable Limits: LEL: N/A **UEL:** N/A
Extinguishing Media: Carbon dioxide, dry chemical, foam
Special Fire Fighting Procedures: N/A
Unusual Fire and Explosion Hazards: N/A

30/07/57

5- REACTIVITY DATA

Stability: Stable

Conditions to Avoid: Avoid temperatures above 150°F.

Incompatibility (Material to Avoid): Strong oxidizing agents or acids

Hazardous Decomposition Byproducts: Oxides of carbon

Hazardous Polymerization: Not occur

6- HEALTH HAZARD DATA

Routes of Entry:

Skin: N/A

Eyes: Mild irritation

Inhalation: Low or mild irritation.

Carcinogenicity - **NTP:** No

IARC Monographs: No **OSHA Regulated Carcinogen:** No

7- EMERGENCY FIRST AID PROCEDURES

Skin: Wash skin with soap and water.

Eyes: Flush with water for 15 minutes. If irritation persists, seek medical attention

Inhalation: Remove to fresh air. If irritation persists, seek medical attention.

Ingestion: Do not induce vomiting. Give large amounts of water. Seek medical attention.

8- PRECAUTIONS FOR SAFE HANDLING & USE

Steps to be done in case material is released or spilled: Ventilate an area. Wipe up or rinse with tap water, sand or sweeping compound.

Waste Disposal Method: Dispose of in accordance with the state, And put in trash.

Precautions to be taken in handling and storing: Store product in original sealed container at room temperature.

Other Precautions : Keep out of reach of the children. Do not ingest. Avoid skin and eye contact. Avoid contamination of food.

9- CONTROL MEASURES

Respiratory Protection : Use in a well-ventilated area.

VENTILATION:

Local Exhaust: N/A

Mechanical (General): Should be sufficient.

Protective Gloves: Butyl rubber, nitrile rubber, polyethylene or double-gloved latex.

Eyes Protection: Safety glasses.

Work/Hygiene Practices: Handle in accordance with good personal hygiene and safety practices. These practices include avoiding unnecessary exposure.

10- TRANSPORTATION INFORMATION

Not regulated by DOT,IATA or IMO.

30/07/57

Mupirocin Ointment: Concise Prescribing Info

Manufacturer
Charoon Bhesaj

Distributor
Charoon Bhesaj

Mailing Address:
776/2-4 Ratchadapisek Rd.,
Daokanong, Thonburi Bangkok 10600
Contact Information:
Tel: (+66) 2477 3000
Fax: (+66) 2477 3001
Email: crb@crb.co.th
Website: http://www.crb.co.th

Contents
Mupirocin
Indications / Uses: Treatment of various skin infections due to susceptible organisms eg, *Staph aureus*, β-hemolytic streptococcus, *S. pyogenes*.
Dosage / Direction for Use: Apply to the affected area tid for up to 10 days.
Contraindications: Hypersensitivity.
Side Effects / Adverse Reactions: Burning, stinging, itching.

Preg Safety (US)

A	B	C	D	X

Category B: Either animal-reproduction studies have not demonstrated a foetal risk but there are no controlled studies in pregnant women or animal-reproduction studies have shown an adverse effect (other than a decrease in fertility) that was not confirmed in controlled studies in women in the 1st trimester (and there is no evidence of a risk in later trimesters).

Topical Antibiotics
ATC Classification
D06AX09 - mupirocin ; Belongs to the class of other topical antibiotics used in the treatment of dermatological diseases.

Thai FDA Category
E

Presentation / Packing

Form	Packing/Price
Bacidal oint 2 % w/w	5 g x 12 × 1's<br>15 g x 12 × 1's

License Number: ฆท.2284/2559

Sterile Irrigation Solutions: Concise Prescribing Info

Manufacturer
A.N.B. Laboratories Co., Ltd.
557 Ramintra Rd., Kannayao, Bangkok, Thailand 10230 / Tel: (+662) 5100021, 9431504-8 / Fax: (+662) 5109945, 5190421

Distributor
Delfi Marketing

Contents
Sodium chloride.
Indications / Uses: Wound irrigation, nasal irrigation, facial cleansing and contact lens rinsing.
Special Precautions: For Irrigation. Not for injection.

Preg Safety (US)
A B **C** D X
Category C: Either studies in animals have revealed adverse effects on the foetus (teratogenic or embryocidal or other) and there are no controlled studies in women or studies in women and animals are not available. Drugs should be given only if the potential benefit justifies the potential risk to the foetus.

Caution For Usage
Instruction for Use: 1. Unscrew the cap and discard the green plastic ring. 2. Screw in the cap tightly until the cone pin punctures through the inner cap and breaks the seal.
The cone pin will also reseal the opened bottle. Single use only. Discard unused portion.
Do not use if seal is damaged.

Storage
Store below 30°C.

Description
Each 100 mL contains: Sodium Chloride 0.9 g. Water for Injection q.s.
Sterile Isotonic Solution. No bacteriostatic present.
Intravenous & Other Sterile Solutions / Surgical Dressings & Wound Care / Nasal Decongestants & Other Nasal Preparations

ATC Classification
V07AB - Solvents and diluting agents, incl. irrigating solutions; Used as solvents and diluting agents, including irrigating solutions.

Regulatory Classification
NP

Presentation/ Packing
Irrigation soln 0.9% x 100 mL, 500 mL, 1,000 mL.

Appendix-II Participants' information sheet and informed consent form (in Thai).

Participant Information Sheet

ข้อมูลสำหรับผู้ป่วยหรืออาสาสมัคร (อายุ 18 ปีขึ้นไป)

ชื่อโครงการศึกษาวิจัย: ประสิทธิผลและความปลอดภัยของยาคลอเฮ็กซิดีนชนิดแผ่นผ้าเปรียบเทียบกับยาขี้ผึ้งมิวพิโรซินในการป้องกันการติดเชื้อที่สัมพันธ์กับการล้างไตทางช่องท้อง: การศึกษาทดลองเปรียบเทียบกลุ่มควบคุมแบบสุ่มแบ่งชั้น ชนิดปกปิด 2 ทาง

หมายเลขโครงการศึกษาวิจัย: NCD58007P3

ผู้ให้ทุนสนับสนุนการวิจัย: สถาบันวิจัยระบบสาธารณสุข (สวรส.) ภายใต้เครือข่ายองค์กรบริหารงานวิจัยแห่งชาติ (คอบช.)

ผู้วิจัย: อาจารย์ เภสัชกร สุรพล โนชัยวงศ์

ท่านได้รับการเชื้อเชิญให้เข้าร่วมการศึกษาวิจัยนี้ เนื่องจากท่านเป็นผู้ป่วยที่ได้รับการบำบัดทดแทนไตด้วยการล้างไตทางช่องท้อง ท่านกรุณาอ่านข้อมูลข้างล่างก่อน (หรือผู้วิจัยได้อ่านให้ท่านรับทราบ) หากท่านมีข้อข้องใจใดๆ เกี่ยวกับการศึกษาวิจัยนี้และสิทธิของท่าน กรุณาซักถามจากแพทย์ผู้ทำการศึกษาวิจัย หรือ ผู้ช่วยแพทย์ที่ทำการศึกษาวิจัย ซึ่งจะเป็นผู้สามารถให้ความกระจ่างแก่ท่านได้ หากท่านตัดสินใจเข้าร่วมการศึกษาวิจัย ท่านจะได้รับเอกสารข้อมูลสำหรับผู้เข้าร่วมโครงการวิจัยและสำเนาใบยินยอมที่ท่านเซ็นชื่อกำกับเก็บไว้ 1 ฉบับ

การศึกษาวิจัยนี้เกี่ยวกับเรื่องอะไร
แผ่นผ้าที่มีตัวยาเคลือบชื่อ "คลอเฮ็กซิดีน กลูโคเนต" และยาขี้ผึ้งชื่อ "มิวพิโรซิน" ได้ถูกพัฒนาขึ้นเพื่อใช้ในการป้องกันและลดโอกาสในการติดเชื้อแบคทีเรีย และพบว่าอาจสามารถใช้ป้องกันการติดเชื้อที่สัมพันธ์กับการล้างไตทางช่องท้องได้ ซึ่งเป็นภาวะแทรกซ้อนชนิดหนึ่งที่พบได้บ่อยในผู้ป่วยที่ได้รับการล้างไตทางช่องท้อง ทางผู้วิจัยจึงต้องการศึกษาว่ายาคลอเฮ็กซิดีนชนิดแผ่นผ้า และ ยาขี้ผึ้งมิวพิโรซิน จะสามารถใช้ป้องกันการติดเชื้อที่สัมพันธ์กับการล้างไตทางช่องท้องได้หรือไม่ โดยเปรียบเทียบกับการรักษาตามมาตรฐาน คือ การใช้สารละลายน้ำเกลือนอร์มัล และเทคนิคปลอดเชื้อในการดูแลช่องสายออก การศึกษานี้จะรวบรวมผู้ป่วยประมาณ 267 ราย จาก 3 โรงพยาบาล คือ โรงพยาบาลนครพิงค์ จังหวัดเชียงใหม่ โรงพยาบาลมหาราชนครเชียงใหม่ มหาวิทยาลัยเชียงใหม่ และโรงพยาบาลสงขลานครินทร์ มหาวิทยาลัยสงขลานครินทร์

ท่านจะต้องปฏิบัติตัวอย่างไร
หากท่านตัดสินใจเข้าร่วมการศึกษาวิจัยนี้ท่านจะถูกขอร้องให้เซ็นชื่อลงในใบยินยอม ท่านจะได้รับการตรวจร่างกาย และท่านจะต้องแจ้งให้แพทย์ของท่านทราบว่าในระยะเวลา 2 สัปดาห์ที่ผ่านมา ท่านได้รับยาอะไรบ้าง หรือกำลังรับยาใดอยู่ ก่อนเริ่มเข้าสู่การศึกษา ท่านจะได้รับการเจาะเลือด เพื่อตรวจทางห้องปฏิบัติการพื้นฐานที่จำเป็น นอกจากนี้ท่านและผู้ดูแลท่าน (ผู้ช่วยในการเปลี่ยนถ่ายน้ำยาล้างไต) จะได้รับการตรวจวัดปริมาณเชื้อแบคทีเรียที่บริเวณโพรงจมูกและรอบทางออกสาย เพื่อใช้ประเมินความเสี่ยงในการติดเชื้อแบคทีเรียที่สัมพันธ์กับการล้างไตทางช่องท้องในอนาคต ทั้งนี้ท่านจะสามารถเริ่มได้รับยาคลอเฮ็กซิดีนชนิดแผ่นผ้า ยาขี้ผึ้งมิวพิโรซิน หรือการรักษามาตรฐาน ก็ต่อเมื่อผลการตรวจ/สัมภาษณ์ยืนยันว่าท่านเหมาะสมที่จะเข้าร่วมการศึกษาวิจัย

เมื่อท่านยินดีเข้าร่วมการศึกษาวิจัย ท่านอาจจะได้รับมาตรการดังต่อไปนี้
1) ยาคลอเฮ็กซิดีนชนิดแผ่นผ้า เช็ดทำความสะอาดรอบทางออกสายวันละครั้ง ร่วมกับขี้ผึ้งหลอกซึ่งไม่มีตัวยา และสารละลายน้ำเกลือนอร์มัล ในการทำความสะอาดทางออกสาย

หรือ 2) ยาขี้ผึ้งมิวพิโรซิน ป้ายรอบทางออกสายวันละครั้ง ร่วมกับแผ่นผ้าหลอกซึ่งไม่มีตัวยา และสารละลายน้ำเกลือนอร์มัล ในการทำความสะอาดทางออกสาย

หรือ 3) การรักษามาตรฐาน คือ สารละลายน้ำเกลือนอร์มัล เช็ดทำความสะอาดรอบทางออกสายวันละครั้ง ร่วมกับแผ่นผ้าหลอกซึ่งไม่มีตัวยา และขี้ผึ้งหลอกซึ่งไม่มีตัวยา ในการทำความสะอาดทางออกสาย

โดยท่านจะได้รับยาคลอเฮ็กซิดีนชนิดแผ่นผ้า ยาขี้ผึ้งมิวพิโรซิน หรือการรักษามาตรฐาน ในจำนวนเพียงพอจนกว่าท่านจะมาพบแพทย์ครั้งต่อไป ท่านจะได้รับการประเมินความร่วมมือในการใช้ยา ทั้งในระหว่างที่มาตรวจรับบริการทางการแพทย์ และการสัมภาษณ์ทางโทรศัพท์ตลอดระยะเวลาที่เข้าร่วมโครงการ นอกจากนี้ท่านจะต้องนำซองยาทุกซอง และผลิตภัณฑ์ที่เหลือ (แผ่นผ้า และยาขี้ผึ้ง) มาด้วยทุกครั้งที่มาพบแพทย์ เพื่อประเมินความร่วมมือในการใช้ยา

ในระหว่าง 1 เดือนแรกที่เริ่มเข้าสู่โครงการ ท่านจะได้รับการติดต่อทางโทรศัพท์จากผู้วิจัยทุกสัปดาห์ รวมทั้งสิ้นจำนวน 4 ครั้ง และในกรณีที่จำเป็นท่านอาจจะได้รับการติดต่อทางโทรศัพท์เพิ่มเติมภายหลัง ระยะเวลาทั้งหมดที่ใช้ในการศึกษา คือ 24 เดือน นับแต่ที่ท่านได้รับยาที่ใช้ในการศึกษา โดยท่านจะต้องมาพบแพทย์รวมทั้งสิ้น 8 ครั้ง ตลอดระยะเวลาที่เข้าร่วมการศึกษา แต่ละครั้งที่ท่านมาพบแพทย์ท่านจะได้รับการเจาะเลือด ประมาณ 10 มิลลิลิตร คิดเป็นปริมาตรทั้งสิ้นประมาณ 40 มิลลิลิตร (ประมาณครึ่งถ้วยตวง) นอกจากนี้ท่านจะถูกซักถามเกี่ยวกับความร่วมมือในการใช้ยา พร้อมกันนั้นแพทย์จะตรวจสุขภาพโดยทั่วไปของท่านด้วย และในกรณีที่จำเป็นท่านอาจจะถูกเก็บตัวอย่างอื่นๆ เพิ่มเติม เช่น การส่งตรวจน้ำยาล้างไต หรือหนอง/สารคัดหลั่งรอบทางออกสาย เพื่อยืนยันการวินิจฉัยโรคในกรณีที่สงสัยการติดเชื้อที่สัมพันธ์กับการล้างไตทางช่องท้อง

ท่านไม่ควรจะรับประทานยาหรือใช้ยาอื่นร่วมด้วย หากแพทย์ของท่านไม่เห็นด้วย แต่หากมีเหตุจำเป็นท่านต้องแจ้งให้แพทย์ของท่านทราบ หากท่านลืมรับประทานยาหรือใช้ยาไม่ว่าด้วยเหตุผลใด หรือท่านรู้สึกไม่สบาย หรือ รู้สึกแตกต่างจากการมาพบแพทย์ครั้งก่อน ขอให้ท่านแจ้งต่อแพทย์ของท่านด้วย

หากท่านไปพบแพทย์ท่านอื่นเพื่อรับการรักษา ขอให้ท่านแจ้งต่อแพทย์ท่านนั้นด้วยว่าท่านกำลังร่วมการศึกษาวิจัยนี้อยู่ ท่านจะได้รับบัตรที่บ่งบอกถึงการศึกษาวิจัยนี้ และท่านควรจะพกบัตรนี้ติดตัวตลอดเวลาเพื่อที่จะแสดงต่อพยาบาล หรือแพทย์ท่านอื่น

ความเสี่ยงจากการเข้าร่วมการวิจัยนี้

ผลข้างเคียงจากยา

ปัจจุบันมีการใช้ยาขี้ผึ้งมิวพิโรซิน หรือสารละลายน้ำเกลือนอร์มัล ในผู้ป่วยที่ได้รับการล้างไตทางช่องท้องอยู่แล้ว ในขณะที่ยาคลอเฮ็กซิดีนแม้จะยังไม่มีการใช้ในผู้ป่วยที่ได้รับการล้างไตทางช่องอย่างแพร่หลาย แต่ก็มีการนำมาใช้ในผู้ป่วยที่เข้ารับการรักษาตัวในโรงพยาบาลเพื่อป้องกันการติดเชื้อแบคทีเรีย โดยทั่วไปผู้ป่วยสามารถทนต่อยาทั้ง 3 ชนิดข้างต้นได้ดี ผลข้างเคียงส่วนใหญ่ไม่รุนแรง และเกิดกับระบบผิวหนังเป็นหลัก เช่น ระคายเคือง ผื่นผิวหนัง หรืออาการแพ้ทางผิวหนัง มีส่วนน้อยที่เกิดอาการรุนแรง คือ ภาวะฉุกเฉินที่เกิดจากปฏิกิริยาภูมิแพ้อย่างเฉียบพลัน หรือการระคายเคืองอย่างรุนแรง ซึ่งมักเกิดในกรณีที่สัมผัสกับตัวยาในความเข้มข้นสูงโดยตรง

ความเสี่ยงจากการเจาะเลือด

ท่านอาจรู้สึกหน้ามืด เป็นลม ปวดบริเวณที่เจาะ หรือมีจ้ำเลือดบริเวณที่เจาะ มีความเสี่ยงน้อยมากที่จะเกิดการติดเชื้อจากการเจาะเลือดเพราะเข็มที่ใช้นั้น ปราศจากเชื้อและใช้ครั้งเดียวทิ้ง

ท่านจะได้ประโยชน์อะไรจากการศึกษานี้

ท่านอาจสามารถลดความเสี่ยงในการเกิดภาวะแทรกซ้อนจากการล้างไตทางช่องท้อง คือ การติดเชื้อที่สัมพันธ์กับการล้างไตทางช่อง และเพิ่มความสำเร็จในการรักษาด้วยการล้างไตทางช่องท้อง นอกจากนี้ผลสรุปที่ได้จากการศึกษานี้จะเป็นประโยชน์ต่อผู้ป่วยรายอื่นในอนาคต

ค่าใช้จ่ายในการเข้าร่วมวิจัย

ทางคณะผู้วิจัย จะเป็นผู้ออกค่าใช้จ่ายในส่วนที่เกี่ยวข้องกับยาที่ใช้ในการศึกษาวิจัย และค่าเดินทาง/ค่าเสียโอกาสที่เกิดขึ้นในการมาตรวจรับบริการ ส่วนค่าตรวจทางห้องปฏิบัติการอื่นที่เกี่ยวข้อง เป็นค่าใช้จ่ายที่เกิดขึ้นจากขั้นตอนการรักษาปกติ ท่านจะต้องเป็นผู้รับผิดชอบ ในกรณีที่ท่านมีสิทธิเบิกค่ารักษา กรุณาสอบถามสิทธิของท่านจากแพทย์ผู้วิจัย

ค่าตอบแทน

ท่านจะได้รับค่าเดินทาง/ค่าเสียโอกาส จากการเข้าร่วมการศึกษาวิจัยนี้ เหมาจ่าย 500 บาท/ครั้ง ที่ท่านมาพบแพทย์ คิดเป็น 4,000 บาทตลอดระยะเวลาที่เข้าร่วมโครงการ 24 เดือน

หากท่านได้รับบาดเจ็บจากการเข้าร่วมการศึกษาวิจัย

หากท่านได้ปฏิบัติตามคำแนะนำของแพทย์ผู้วิจัยแล้ว และยาที่ใช้ในการศึกษาวิจัยหรือกระบวนการต่าง ๆ ในการวิจัยทำให้ท่านได้รับบาดเจ็บ แพทย์ผู้รักษาจะดำเนินการรักษาพยาบาลให้แก่ท่านตามแนวทางการรักษามาตรฐาน ตามสิทธิผู้ป่วยที่ท่านพึงมี

ทางเลือกอื่นในการป้องกันการติดเชื้อที่สัมพันธ์กับการล้างไตทางช่องท้อง

ท่านไม่จำเป็นต้องเข้าร่วมการศึกษาวิจัยเพื่อให้ได้รับมาตรการในการป้องกันการติดเชื้อที่สัมพันธ์กับการล้างไตทางช่องท้อง ปัจจุบันแม้ยังไม่มียามาตรฐานที่ใช้ในการป้องกันการติดเชื้อที่สัมพันธ์กับการล้างไตทางช่องท้อง แต่แนวทางการรักษาปกติของของโรงพยาบาลที่ท่านใช้บริการอยู่ เช่น เทคนิคปลอดเชื้อในการดูแลทางออกสาย หรือการส่งเสริมภาวะโภชนาการที่เหมาะสม ก็สามารถป้องกันการติดเชื้อที่สัมพันธ์กับการล้างไตทางช่องท้องได้ดีในระดับหนึ่ง

ท่านจะทำอย่างไรหากท่านไม่ต้องการเข้าร่วมการศึกษาวิจัย หรือเปลี่ยนใจระหว่างร่วมศึกษาวิจัย

ท่านไม่จำเป็นต้องเข้าร่วมการศึกษาวิจัยนี้หากท่านไม่สมัครใจ หลังจากท่านตัดสินใจจะเข้าร่วมการศึกษาแล้ว ท่านสามารถจะถอนตัวได้ตลอดเวลา การตัดสินใจของท่านจะไม่มีผลต่อการรักษาในอนาคต หรือการดูแลอื่นใด หากท่านไม่ต้องการเข้าร่วมการศึกษาหรือต้องการหยุดการศึกษา ณ เวลาใดก็ตาม

ในกรณีที่มีแนวทางการป้องกันการติดเชื้อที่สัมพันธ์กับการล้างไตทางช่องท้องที่มีประสิทธิภาพดีกว่า คณะผู้วิจัยอาจจะตัดสินใจยกเลิกท่านจากการศึกษา หากเห็นว่าจะเป็นประโยชน์สำหรับท่านมากกว่า

ใครจะรู้บ้างว่าท่านเข้าร่วมการศึกษานี้

แพทย์ประจำตัวท่าน (แพทย์เวชปฏิบัติทั่วไป) ควรจะได้รับทราบว่าท่านตัดสินใจเข้าร่วมการศึกษาวิจัยนี้ ข้อมูลของท่านที่ถูกบันทึกไว้ระหว่างการศึกษา เช่นเดียวกับข้อมูลที่เกี่ยวข้องจากแฟ้มเวชระเบียนของโรงพยาบาล คลินิก หรือข้อมูลอื่น ๆ จะถูกเก็บไว้เป็นความลับตลอดเวลา คณะกรรมการจริยธรรมการวิจัย และคณะผู้วิจัยหรือผู้แทนที่คณะผู้วิจัยได้จัดตั้งขึ้น สามารถที่จะขอตรวจสอบข้อมูลเหล่านี้ได้ โดยข้อมูลเหล่านี้จะยังเก็บรักษาไว้เป็นเรื่องลับเฉพาะ

การปกป้องรักษาข้อมูล: ข้อมูลใดบ้างที่จะถูกเก็บรวบรวมไว้จากการศึกษานี้

ข้อมูลส่วนตัวที่ท่านไม่ต้องการเปิดเผยจะถูกเก็บรวบรวมไว้และนำมาใช้เพื่อวัตถุประสงค์ทางการวิจัยทางการแพทย์ เฉพาะในส่วนที่เกี่ยวข้องกับการศึกษา ข้อมูลดังกล่าวจะถูกสรุปผลในภาพรวม โดยจะไม่มีการอ้างถึงชื่อท่านในรายงานหรือวารสารใด ๆ

หากท่านตกลงใจเข้าร่วมการศึกษา ท่านยินยอมที่จะไม่จำกัดการให้ข้อมูลที่เป็นส่วนตัว ยกเว้นในกรณีที่ขัดต่อสิทธิส่วนบุคคลภายใต้ (ก) ข้อบังคับแพทยสภาว่าด้วยการรักษาจริยธรรมแห่งวิชาชีพเวชกรรม พ.ศ. 2549 หมวด 9 การศึกษาวิจัยและการทดลองในมนุษย์ และ (ข) สิทธิผู้ป่วยของ แพทยสภา, สภาการพยาบาล, ทันตแพทยสภา บุคคลอื่นในนามของคณะผู้วิจัยอาจจะได้รับอนุญาตให้เข้ามาตรวจและประมวลข้อมูลส่วนตัวของท่าน ทั้งนี้จะต้องกระทำไปตามคำบอกกล่าวของคณะผู้วิจัย และเพื่อวัตถุประสงค์ทางการศึกษาวิจัยทางการแพทย์เท่านั้น โดยคณะผู้วิจัยจะทำทุกวิถีทางเพื่อให้เกิดความมั่นใจว่าข้อมูลส่วนตัวของท่านจะถูกปกป้องไว้

หากสิ้นสุดการศึกษาแล้วยาที่ใช้ในการศึกษาจะมีให้ท่านหรือไม่

ภายหลังสิ้นสุดการศึกษา แพทย์ของท่านจะพิจารณาตามแนวทางปกติของแต่ละสถาบัน/โรงพยาบาล ในการป้องกันการติดเชื้อที่สัมพันธ์กับการล้างไตทางช่องท้อง ซึ่งอาจทำให้ท่านได้รับยาขี้ผึ้งมิวพิโรซิน หรือสารละลายน้ำเกลือนอร์มัล และเทคนิคปลอดเชื้อในการดูแลทางออกสาย ในกรณียาคลอเฮ็กซิดีนชนิดแผ่นผ้านั้น หากพบว่ามีความประสิทธิภาพ ความปลอดภัย และความคุ้มค่าในการใช้ ผู้วิจัยจะขยายผลโดยการเผยแพร่และส่งเสริมมาตรการดังกล่าวนี้ เพื่อให้เกิดการใช้อย่างแพร่หลายต่อไป

หากท่านมีคำถามเกี่ยวกับการศึกษานี้ท่านสามารถติดต่อใครได้บ้าง

หากท่านมีคำถามหรือมีความวิตกกังวลเกี่ยวกับการศึกษาวิจัยนี้ หรือสงสัยว่าท่านกำลังได้รับบาดเจ็บจากการเข้าร่วมการวิจัยนี้ กรุณาติดต่อ เภสัชกร สุรพล โนชัยวงศ์ เบอร์โทรศัพท์ 053-944-334 (ในเวลาราชการ) โทรศัพท์เคลื่อนที่ 089-9973365 (ทั้งในและนอกเวลาราชการ) หรือติดต่อที่ ภาควิชาบริบาลเภสัชกรรม คณะเภสัชศาสตร์ มหาวิทยาลัยเชียงใหม่ ถ.สุเทพ ต.สุเทพ อ.เมือง จ.เชียงใหม่ 50200 โทรศัพท์ 0-5394-4334 (ในเวลาราชการ) โทรสาร 0-5322-2741 (ในเวลาราชการ)

INFORMED CONSENT FORM

หนังสือแสดงความยินยอมการเข้าร่วมในโครงการวิจัย
สำหรับอาสาสมัคร อายุ 18 ปีขึ้นไป

ข้าพเจ้า นาย/นาง/นางสาว .... ขอให้ความยินยอมของตนเองที่จะเข้าร่วมในการศึกษาวิจัยเรื่อง “ประสิทธิผลและความปลอดภัยของยาคลอเฮกซิดีนชนิดแผ่นผ้าเปรียบเทียบกับยาขี้ผึ้งมิวพิโรซินในการป้องกันการติดเชื้อที่สัมพันธ์กับการล้างไตทางช่องท้อง: การศึกษาทดลองเปรียบเทียบกลุ่มควบคุมแบบสุ่มแบ่งชั้น ชนิดปกปิด 2 ทาง”

ข้าพเจ้าได้รับข้อมูลและคำอธิบายเกี่ยวกับการวิจัยนี้แล้ว ข้าพเจ้าได้มีโอกาสซักถามเกี่ยวกับการวิจัยนี้และได้รับคำตอบเป็นที่พอใจแล้ว ข้าพเจ้ามีเวลาเพียงพอในการอ่านและทำความเข้าใจกับข้อมูลในเอกสารนี้อย่างถี่ถ้วน และได้รับเวลาเพียงพอในการตัดสินใจว่าจะเข้าร่วมการศึกษาวิจัยนี้หรือไม่ ผู้วิจัยมีความยินดีที่จะให้คำตอบต่อคำถามประการใดที่ข้าพเจ้าอาจจะมีได้ ตลอดระยะเวลาการเข้าร่วมการวิจัยครั้งนี้ ผู้วิจัยรับรองว่าจะเก็บข้อมูลเฉพาะที่เกี่ยวกับตัวข้าพเจ้าเป็นความลับ และจะเปิดเผยได้เฉพาะในรูปที่เป็นสรุปผลการวิจัย และผู้วิจัยจะได้ปฏิบัติในส่งที่ไม่ก่อให้เกิดอันตรายต่อร่างกาย หรือจิตใจของข้าพเจ้าตลอดการวิจัยนี้ และรับรองว่าหากเกิดมีอันตรายใดๆ จากการวิจัยดังกล่าว ผู้ยินยอมจะได้รับการรักษาอย่างเต็มที่

ข้าพเจ้ายินยอมเข้าร่วมการวิจัยโดยสมัครใจ และสามารถที่จะถอนตัวจากการวิจัยนี้เมื่อใดก็ได้ ทั้งนี้ โดยไม่มีผลกระทบต่อการรักษาพยาบาลที่ข้าพเจ้าจะได้รับถ้าหากข้าพเจ้าเป็นผู้ป่วย และในกรณีที่เกิดข้อข้องใจหรือปัญหาที่ข้าพเจ้าต้องการปรึกษากับผู้วิจัย ข้าพเจ้าสามารถติดต่อกับผู้วิจัย คือ อาจารย์ เภสัชกร สุรพล โนชัยวงศ์ ได้ที่ ภาควิชาบริบาลเภสัชกรรม คณะเภสัชศาสตร์ มหาวิทยาลัยเชียงใหม่ ถ.สุเทพ ต.สุเทพ อ.เมือง จ.เชียงใหม่ 50200 โทรศัพท์ที่ทำงาน 053-944-334 โทรศัพท์เคลื่อนที่ 089-997-3365 โทรสาร 053-222-741

โดยการลงนามนี้ ข้าพเจ้าไม่ได้สละสิทธิ์ใดๆ ที่ข้าพเจ้าพึงมีทางกฎหมาย

ลายมือชื่ออาสาสมัคร ________________________________ วัน-เดือน-ปี______________
(________________________________)

ลายมือชื่อผู้ดูแล/ญาติ ________________________________ วัน-เดือน-ปี______________
(________________________________)

ลายมือชื่อผู้ให้ข้อมูลการวิจัย ____________________________ วัน-เดือน-ปี______________
(________________________________)

พยาน ________________________________ วัน-เดือน-ปี______________
(________________________________)
